# The Endosome Localized Arf-GAP AGAP1 Modulates Dendritic Spine Morphology Downstream of the Neurodevelopmental Disorder Factor Dysbindin

**DOI:** 10.3389/fncel.2016.00218

**Published:** 2016-09-22

**Authors:** Miranda Arnold, Rebecca Cross, Kaela S. Singleton, Stephanie Zlatic, Christopher Chapleau, Ariana P. Mullin, Isaiah Rolle, Carlene C. Moore, Anne Theibert, Lucas Pozzo-Miller, Victor Faundez, Jennifer Larimore

**Affiliations:** ^1^Department of Biology, Agnes-Scott CollegeDecatur, GA, USA; ^2^Interdisciplinary Program in Neuroscience, Georgetown UniversityWashington, DC, USA; ^3^Department of Cell Biology, Emory UniversityAtlanta, GA, USA; ^4^Department of Neurobiology, Civitan International Research Center, University of AlabamaBirmingham, AL, USA; ^5^Acorda TherapeuticsNew York, NY, USA; ^6^Heritage College of Osteopathic Medicine, The Medical School of Ohio UniversityAthens, OH, USA

**Keywords:** AGAP1, Arf-GAP, autism, dendritic spines, dysbindin, endosome, schizophrenia

## Abstract

AGAP1 is an Arf1 GTPase activating protein that interacts with the vesicle-associated protein complexes adaptor protein 3 (AP-3) and Biogenesis of Lysosome Related Organelles Complex-1 (BLOC-1). Overexpression of AGAP1 in non-neuronal cells results in an accumulation of endosomal cargoes, which suggests a role in endosome-dependent traffic. In addition, AGAP1 is a candidate susceptibility gene for two neurodevelopmental disorders, autism spectrum disorder (ASD) and schizophrenia (SZ); yet its localization and function in neurons have not been described. Here, we describe that AGAP1 localizes to axons, dendrites, dendritic spines and synapses, colocalizing preferentially with markers of early and recycling endosomes. Functional studies reveal overexpression and down-regulation of AGAP1 affects both neuronal endosomal trafficking and dendritic spine morphology, supporting a role for AGAP1 in the recycling endosomal trafficking involved in their morphogenesis. Finally, we determined the sensitivity of AGAP1 expression to mutations in the *DTNBP1* gene, which is associated with neurodevelopmental disorder, and found that AGAP1 mRNA and protein levels are selectively reduced in the null allele of the mouse ortholog of *DTNBP1*. We postulate that endosomal trafficking contributes to the pathogenesis of neurodevelopmental disorders affecting dendritic spine morphology, and thus excitatory synapse structure and function.

## Introduction

ADP-ribosylation factors (Arfs) are members of the Ras superfamily of GTPases that participate in membrane trafficking along the secretory and endosomal pathways (Powelka and Buckley, 2001; Bader et al., 2004; Randazzo and Hirsch, 2004; D’Souza-Schorey and Chavrier, 2006; Nie and Randazzo, 2006; Wang et al., 2006). Arf GTP binding is catalyzed by GTPase activating proteins (GAPs; Donaldson and Jackson, 2000; Nie and Randazzo, 2006). Arf1 GTPase activating protein (AGAP1) is a member of the AZAP family of phosphoinositide-regulated Arf GAPs, whose members contain a conserved Arf GAP domain, pleckstrin homology (PH) domain and ankyrin repeats (Nie et al., 2002). AZAPs regulate membrane traffic by specifying the time and place of coat recruitment to generate nascent vesicles and possibly as part of the coat during vesicle budding (Randazzo and Hirsch, 2004).

In this study, we focus on AGAP1 (also called GGAP, KIAA1099 and centaurin γ-2/CENTG2), which has been primarily described in non-neuronal cells, where AGAP1 up-regulation results in impaired endosomal trafficking (Nie et al., 2002). AGAP1 associates with the endosomal complexes adaptor protein 3 (AP-3) and Biogenesis of Lysosome Related Organelles Complex-1 (BLOC-1: Nie et al., 2005; Bendor et al., 2010). In neuronal cells, AGAP1 is necessary for the recycling of muscarinic acetylcholine receptors in association with the AP-3 adaptor and with dysbindin, a subunit of the BLOC-1 (Bendor et al., 2010). Diverse evidence suggests that AGAP1, AP-3, and BLOC-1 subunits are risk factors in neurodevelopmental disorders. The BLOC-1 subunit dysbindin encoded by the gene *DTNBP1* is a risk factor for schizophrenia (SZ) and autism spectrum disorder (ASD; Tang et al., 2009; Greenwood et al., 2011; Di Benedetto et al., 2013); indeed, the dysbindin levels are lower in the brains of individuals with SZ (Talbot et al., 2004). The neuronal specific AP-3 subunit AP-3 beta2 is also a risk factor for ASD (O’Roak et al., 2012). Furthermore, AGAP1 is a candidate ASD susceptibility gene (Wassink et al., 2005; Sebat et al., 2007), and has been reported as a potential risk factor in a genome-wide association studies (GWAS) of SZ (Shi et al., 2009). These data support the hypothesis that impaired mechanisms of AGAP1-dependent endosomal trafficking contribute to the pathogenesis of neurodevelopmental disorders such as ASD and SZ (Ryder and Faundez, 2009). However, AGAP1 localization, function, and susceptibility to mutations in genes associated with neurodevelopmental disorders have not been explored in neurons.

We examined the expression levels and subcellular localization of endogenous AGAP1 in primary neurons, and investigated the consequences of altered AGAP1 levels on endosomal trafficking and dendritic spine morphology. AGAP1 is expressed in neurons of the developing brain, localizing to axons, dendrites, and dendritic spines that receive excitatory synapses, where colocalizes with endosomal markers. Overexpression or down-regulation of AGAP1 levels alter the recycling of endosome cargoes in dendrites, and alters spine density. In addition, AGAP1 is downstream of the BLOC-1 subunit dysbindin, the ortholog of the human SZ susceptibility gene *DTNBP1*. Indeed, mRNA and protein levels of AGAP1 are lower in the hippocampus of mice carrying null mutations in dysbindin (*Bloc1s8^sdy/sdy^*) compared to their wild type (WT) littermates. Together, these data support the hypothesis that AGAP1 regulates endosomal trafficking and dendritic spine morphology during early brain development downstream of a gene implicated in the pathogenesis of SZ.

## Materials and Methods

### Plasmids

eGFP and yellow fluorescent protein (YFP) vectors were purchased from Clontech (Mountain View, CA, USA). WT AGAP-1 vectors were kindly supplied by Dr. Nie (NIH). siRNA sequences were selected using siRNA Design SciTool (Integrated DNA Technologies, Coralville, IA, USA), and Blast analysis comparing human and rat AGAP1. The 4411 sequence 5′CUACCAUUAA UCUGAAAAAC3′ and the 3890 sequence 5′CCUUCCCAAAUUCACUGUC3′ were purchased from Integrated DNA Technologies (Coralville, IA, USA).

### Antibodies

The polyclonal rabbit antibody against the C-terminus of AGAP1 was previously characterized, and shown to be specific for AGAP1 (Nie et al., 2002, 2003, 2005). It recognizes both human and rodent AGAP1, is specific for AGAP1, and does not cross react with AGAP2 or other proteins (Nie et al., 2002, 2003; Xia et al., 2003). The polyclonal rabbit anti-AGAP1 used in primary cultures was a gift from Dr. Nie; this antibody was compared to the rabbit anti-AGAP1 purchased from Sigma, and found to be specific for the C-terminus of AGAP1. The Sigma rabbit anti-AGAP1 was used in sections of perfusion-fixed brains. Mouse anti-synaptotagmin (Syt1) and mouse anti-synaptophysin antibodies were purchased from Chemicon International, Inc. (Billerica, MA, USA). Goat anti-Neurabin II (A-20), mouse anti-MAP-2, mouse anti-EEA1, and mouse anti-transferrin receptor antibodies were purchased from Zymed Laboratories Inc. (San Francisco, CA, USA). Mouse anti-PSD-95 clone K28/43 was purchased from Upstate Biotechnology (Lake Placid, NY, USA). Mouse anti-GAPDH, goat anti-chromogranin B, mouse anti-GM130, Texas Red, Cascade blue, FITC-conjugated anti-rabbit and anti-mouse secondary antibodies, and HRP-conjugated secondary antibodies were purchased from Santa Cruz (Santa Cruz, CA, USA). Texas-Red-conjugated anti-mouse and anti-rabbit secondary antibodies were purchased from Vector Laboratories, Inc. (Burlingame, CA, USA). Mouse anti-β-tubulin and mouse anti-actin were purchased from the Developmental Studies Hybridoma Bank University of Iowa (Iowa City, IA, USA). Mouse anti-Tau was a gift from Dr. Gail Johnson (University of Alabama at Birmingham). Rabbit anti-VAMP-2 was purchased from Synaptic Systems (Göttingen, Germany).

### Mice

*Bloc1s8^sdy/sdy^* (*sandy*) mice have the C57/B6 genetic background, and were previously described (Larimore et al., 2014). Mouse genotyping was performed by PCR of genomic DNA as previously described (Larimore et al., 2014). All animal procedures were approved by the IUCAC at Emory University. Mecp2 mice STOCK *Mecp2*^tm1.1Jae^/Mmucd and their controls were a gift from Dr. LP-M (University of Alabama at Birmingham) as previously described (Larimore et al., 2013).

### Immunoblot Analyses

Brain lysates were separated by SDS–PAGE and transferred to PVDF membranes (BioRad, Hercules, CA, USA). Membranes were probed with primary antibodies followed by HRP-conjugated anti-rabbit and anti-mouse secondary antibodies (Santa Cruz Biotechnology, Santa Cruz, CA, USA). Secondary antibodies were detected using Supersignal West Dura Extended Duration substrate (Pierce Chemical, Rockford, IL, USA) and film developing.

### Synaptosome Preparation

Synaptosomes were prepared from postnatal-day (P) 23 WT mice, as previously described (Larimore et al., 2011). Briefly, mice were anesthetized by CO_2_ inhalation, and their brains quickly transferred to ice-cold phosphate-buffered saline (PBS). Tissue was homogenized by 16 strokes of a Potter-Elvehjem homogenizer at 800 rpm in 0.32M sucrose, 5 mM HEPES, and 0.5 mM EDTA, supplemented with complete anti-protease inhibitor (Roche Molecular Biochemicals; Indianapolis, IN, USA). Homogenates were spun at 1000× g for 10 min and S1 supernatants were further sedimented at 12,000× g for 20 min. The resulting P2 pellet was resuspended in 8.5% Percoll (Sigma-Aldrich). The resuspended P2 pellet was then loaded on a discontinuous gradient comprised of 10 and 16% Percoll (Larimore et al., 2011). Gradients were spun at 15,000× g for 20 min.

### Neuronal Cultures and Electroporation Transfections

Hippocampal neurons were cultured form embryonic day (E) 18 Sprague-Dawley rat embryos dissected from pregnant rats (purchased from Charles Rivers Laboratories, Wilmington, MA, USA), as previously described (Larimore et al., 2009). Dissociated neurons were plated on poly-L-lysine (Sigma Aldrich, St. Louis, MO, USA) coated glass cover-slips, and cultured for 3–14 days *in vitro* (DIV). Dissociated neurons were transfected while in suspension using Amaxa nucleofection (Lonza, Walkersville, MD, USA). Dissociated neurons (2 × 10^6^) were transfected with 3 μg plasmid DNA or 3 μg of each siRNA. Medium was added, and neurons were plated at a density of 6 × 10^4^ cells per well in a 12-well plate.

### Immunocytochemistry

Hippocampal neurons on cover-slips were fixed with ice-cold 3.7% formaldehyde in phosphate buffer (PB; 23 mM NaH_2_O_4_, 80 mM Na_2_HPO_4_) for 20 min, permeabilized with ice-cold 3.7% formaldehyde containing 0.25% Triton X-100 for 10 min, washed three times in PBS, and then blocked with 10% bovine serum albumin (BSA) for 1 h at 37°C. Primary and secondary antibodies were diluted in 3% normal horse serum (NHS) in PBS. Cover-slips were incubated in primary antibodies at 4°C overnight, and then washed three times for 10 min at room temperature (RT). Cover-slips were incubated in secondary antibodies for 2 h at RT. Coverslips were washed three times for 10 min each, once with ddH_2_0, and finally mounted on glass slides with Vectashield with or without DAPI (Vector Laboratories, CA, USA). Cover-slips with immunostained neurons were imaged in an epifluorescence inverted microscope (Olympus IX 70) with 40× (X NA) and 100× (X NA) oil-immersion objectives using a monochromatic cooled CCD camera (Retiga 1300, Qimaging, British Columbia, Canada). Images were deconvolved and merged using the microtome extension of IP Lab software (Scanalytics, Rockville, MD, USA), colocalization was measured using the Fluorescence CV extension of IP Lab.

### Transferrin Recycling Assay

Transferrin uptake and trafficking was assayed in neurons as previously described (Prekeris et al., 1999). Hippocampal neurons were transfected using Amaxa nucleofection after dissociation and while in suspension. Neurons were then plated and cultured for 3 DIV, serum starved in imaging buffer for 1 h, and incubated with 50 μg/ml Alexa-transferrin (Alexa-Tf; Molecular Probes, Carlsbad, CA, USA) in 1% BSA for 30 min at 37°C to label the recycling endosome pool. Neurons were washed and incubated with 100 μg/mL unlabeled holo-transferrin (Sigma Aldrich, St. Louis, MO, USA) to allow transferrin to exit the recycling endosome, and then fixed with 4% paraformaldehyde either immediately or after a 45 min chase. Colocalization was measured using Fluorescence CV extension IP Lab, and expressed in percentages for statistical comparisons.

### Organotypic Hippocampal Slice Cultures and Biolistic Transfection

Hippocampal slices were prepared from P7 to P10 Sprague-Dawley rats, and maintained *in vitro* as previously described (Alonso et al., 2004). Slice cultures were transfected by biolistic gene transfer at 6–8 DIV, as described (Boda et al., 2004; Chapleau et al., 2008). Plasmid cDNA encoding enhanced yellow fluorescent protein (eYFP; Clontech, Mountain View, CA, USA) was precipitated onto 1.6-μm diameter colloidal gold (Bio-Rad, Hercules, CA, USA) at a ratio 25 μg DNA eYFP and 45 μg AGAP1–10 mg gold, and coated onto Tefzel tubing, following manufacture’s protocol. For the siRNA, 40 μg of siRNA oligo duplex with 20 μg of eYFP were coated onto 10 mg of gold. Slices were bombarded using a modified Helios gene-gun (Bio-Rad, Hercules, CA, USA; He pressure 100 psi), fixed and mounted on glass slides with Vectashield for confocal imaging 3 days after transfection, as described (Chapleau et al., 2008).

### Confocal Imaging and Analysis of Dendritic Spines

Secondary and tertiary branches of apical dendrites of pyramidal neurons in the CA1 and CA3 region of hippocampal slice cultures were acquired in a laser-scanning confocal microscope (Olympus Fluoview-300, Olympus) using an oil-immersion 100× objective (NA 1.4; Olympus PlanAPo). eYFP was excited using an argon laser (488 nm), and detected using standard FITC filters. Only those pyramidal neurons that displayed eYFP fluorescence throughout the entire dendritic tree and lacked signs of degeneration (i.e., “dendritic blebbing”) were imaged. Series of optical sections in the *z*-axis were acquired at 0.1 μm intervals through each dendritic branch. Microscope calibrations were performed using 1.07 μm latex fluorescent microspheres. Calibrations determined that our effective lateral (*x–y* axis) pixel resolution was 0.092 microns per pixel.

Dendritic spines were identified as small protrusions that extended ≤3 μm from the parent dendrite, and counted off-line using NIH ImageJ as described (Chapleau et al., 2008); spines were counted only if they appeared continuous with the parent dendrite. Numerical density was estimated by quantifying the number of spines in several dendritic segments per neuron, and normalized to 10 μm of dendrite length for statistical comparisons.

Morphological types of spines were defined from ratios of spine length, spine head width and spine neck width, which were measured using ImageJ (NIH), as previously described (Tyler and Pozzo-Miller, 2003; Boda et al., 2004). We manually measured the length (L) and the apparent diameter of spine heads (d_h_) and spine necks (d_n_). Type-I spines (aka stubby) had L~d_n_~d_h_; Type-II spines (aka mushroom) had d_h_ ≫ d_n_; and Type-III spines (aka thin) had L ≫ d_n_.

### Immunohistochemistry and Confocal Microscopy of Brain Sections

Perfusion-fixed brain sections including the hippocampus were processed for fluorescence immunohistochemistry as previously described (Larimore et al., 2011, 2013). Mice under anesthesia were perfused through the heart with lactated ringers followed by 4% glutaraldehyde, brains dissected and sectioned in a vibratome at 60 μm thickness. Freely-floating sections were rinsed with PBS, and incubated in 1% sodium borohydride for 20 min at RT, followed by PBS washes. Sections were blocked with 5% NHS, 1% BSA, and 0.3% Triton X-100 for 60 min at RT. Sections were incubated in primary antibody solutions for 1 h at RT (PBS with 1% NHS and 1% BSA with 1:10,000 dilution of mouse anti-synaptophysin, and 1:100 dilution of rabbit anti-AGAP1). After rinsing in PBS, sections were incubated for 60 min in secondary antibody solutions (PBS with 1% NHS and 1% BSA and 1:500 dilutions); the following Alexa-conjugated secondary antibodies were used: anti-mouse 555 (for anti-Synaptophysin) and anti-rabbit 488 (for anti-AGAP1). Following PBS washes, sections were incubated in cupric sulfate (3.854 W/V Ammonium Acetate, 1.596 W/V Cupric Sulfate in distilled water, pH 5) for 30 min, washed with PBS, and mounted on glass slides with Vectashield. Sections were imaged by laser-scanning confocal microscopy (LSM-510, Axiovert 100M, Zeiss) using 10X/0.5 NA, 20X/0.5 NA dry objectives (Plan Apochromat, Zeiss), and 40X/1.3 NA and 63X/1.4 NA DiC oil objectives. Alexa fluorophores were excited with Argon and HeNe1 lasers, and their emission filtered with BP 505-530 and LP 560. *Z*-stacks were acquired and a minimum of four stacks per hippocampus were analyzed.

### Quantitative Real Time PCR (qtPCR)

qtPCR was performed as previously described (Larimore et al., 2013, 2014). Briefly, the hippocampus of P50 *Bloc1s8^sdy/sdy^* (*sandy*) mice and age-matched WT littermates was dissected and immediately flash-frozen in liquid nitrogen. Following TRIzol (Invitrogen Life Technologies, Grand Island, NY, USA) extraction using SuperScript III First-Strand Synthesis (Invitrogen Life Technologies, Grand Island, NY, USA), isolated mRNA was reverse transcribed into cDNA. PCR amplifications were performed on a LightCycler480 Real Time plate reader using LightCycler 480 SYBR Green reagents (Roche, Indianapolis, IN, USA) at 95°C for 5 min, followed by 45 cycles of 95°C for 5 s, 65°C for 10 s and 72°C for 20 s, followed by a 95°C for 5 s, 65°C for 2 min and a 97°C incubation to determine melting curves. Primers were designed for dysbindin forward gatcgcagagaggcgaga and dysbindin reverse tgagatgtccatcaggtcca; Pallidin forward ctccagacggggtccttac and pallidin reverse agtccttcatctggagacgtg; Vamp2 forward tgcacctcctccaaacctta and Vamp2 reverse agctccgacaacttctggtc; and finally, AGAP1 forward gccgaccagtggagtgac and AGAP1 reverse tacttgggctggaacacacc. PCR amplification was performed in duplicate with a minimum of three WT samples, and three mutant samples. Relative amplification was assigned a concentration value based on standard curves performed prior to the experiments, and normalized to within-plate controls.

## Statistics

Statistical comparisons were made with the engine^1^. Kolmogorov-Smirnov tests were performed with the engine^2^. Means were compared with two-tailed Student’s *t*-tests. The proportions of dendritic spine types were compared by with the Fisher Exact test. *P* values <0.05 were considered statistically significant. Spine density and morphology measurements were done blinded.

## Results

### Endogenous AGAP1 Localizes to Dendritic Endosomes of Hippocampal Neurons

**Figure 1:**
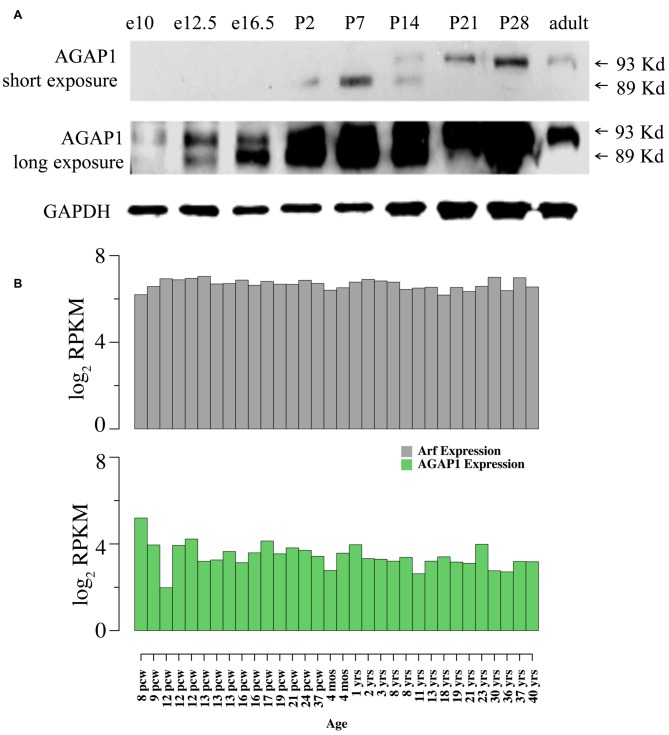
**Developmental expression and neuronal localization of endogenous Arf1 GTPase activating protein (AGAP1). (A)** Immunoblot analysis of AGAP1 protein levels in lysates from whole mouse embryo (E10 and E12.5) and whole mouse brain (E16.5—adult), using a previously characterized specific anti-C terminal peptide antibody (Nie et al., 2002). E16.5 was the first time point that the brain was dissected from other body regions. Thirty Microgram of total protein was loaded in each lane. **(B)** Transcript abundance measured as log2 RPKM (reads per kilobase per million) in developing human hippocampus for the genes ADP-ribosylation factors (Arf1; ENSG00000143761) and AGAP1 (ENSG00000157985). Data were downloaded from http://www.brainspan.org/. Note that expression of Arf1 and AGAP1 transcripts are stable during pre and postnatal development. Post conception week (pcw) (http://www.ncbi.nlm.nih.gov/pubmed/23193282).

We determined expression of AGAP1 during development, AGAP1 was detected as early as E10 in whole embryos, and at E16.5 in brain, increasing during prenatal and postnatal development. Consistent with published observations (Nie et al., 2002), two isoforms of AGAP1 (89 and 93 kDa) were detected in brain using the AGAP1 specific antibody (Figure 1). Interestingly, the two isoforms demonstrated differential developmental expression in brain. The 89 kDa isoform is highly expressed earlier, peaking between P2–P7, whereas the 93 kDa isoform peaks between P14–P21, and persists into adulthood (Figure 1). We analyzed the anatomical distribution of the mouse AGAP1 transcript using the Allen Brain Atlas. The AGAP1 transcript was expressed in all regions, including the hippocampus, thalamus, brain stem, cerebellum, cortex and spinal cord^3^. The hippocampus is a site where expression was among the highest expression areas of the brain. Therefore, we focused our experiments in this anatomical region. We used the Brainspan database and focused on the human hippocampus to assess the developmental expression of AGAP1 and Arf1 transcripts^4^. Arf1 and AGAP1 transcript levels were roughly similar between embryonic and adult hippocampus in clear contrast with transcripts with protein expression in rodent brain (Figure 1B).

**Figure 2:**
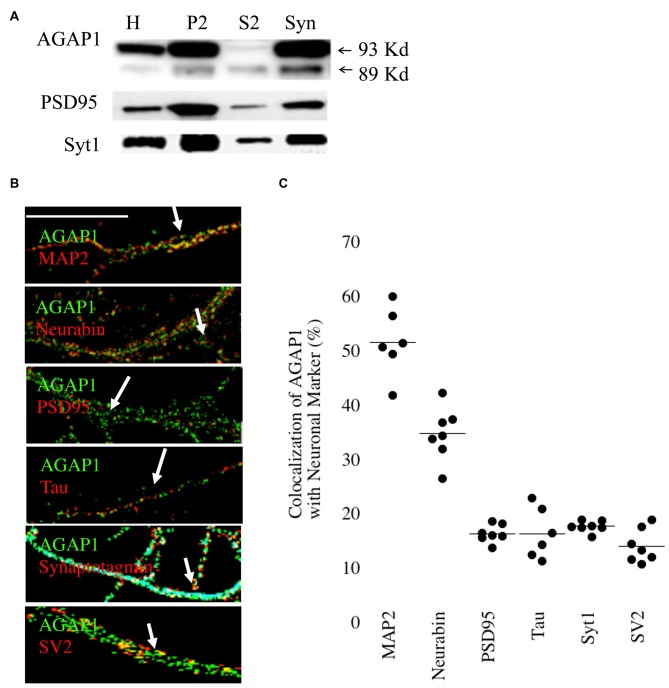
**Synaptic Localization of AGAP1. (A)** Immunoblot analysis of AGAP1 and synaptic markers in fractionated P23 whole rat brain. H-whole brain homogenate, P2-crude microsomes/synaptosomes, S2-supernatant, and Syn-pure synaptosome fraction. Ten micrograms of protein was loaded for each fraction. **(B)** Regions of interest (ROIs) from endogenous immunocytochemistry micrographs imaged from 14 Days *in vitro* (DIV) dissociated rat E18 hippocampal neurons. White arrows indicate regions of colocalization. Green: endogenous AGAP1. Red: MAP-2, neurabin, PSD95, Tau, synaptotagmin (Syt), and SV2 as indicated. Scale bar = 10 μm. **(C)** Quantification of colocalization within ROIs of endogenous AGAP1 and subcellular markers. Data were quantified from three independent experiments (*n* = 10).

Next, we analyzed the subcellular distribution of AGAP1 in cultured hippocampal neurons by subcellular fractionations. We prepared synaptosome-enriched fractions from P21 animals because the majority of synapses have been formed in mouse brain at this age. We detected the two isoforms (89 and 93 kDa) in both the supernatant (S2) and the crude microsome/synaptosome fractions (P2) (Figure 2A), which were described in cell lines (Nie et al., 2002). Further fractionation of the P2 fraction yielded an enriched synaptosome fraction (Syn) containing both presynaptic and postsynaptic compartments, confirmed by the detection of the synaptic vesicle marker Syt, and the postsynaptic density protein PSD95, respectively (Gordon-Weeks, 1987). AGAP1 was enriched in the Syn fraction over the P2 fraction at a level comparable to those of Syt1 and PSD95 (Figure 2A). Our findings are in line with previous data demonstrating first that the Arf that AGAP1 regulates, Arf1, is also localized to the synaptic region (Park et al., 2004, 2006; Kennedy and Ehlers, 2006). Second, our results expand previous biochemical evidence identifying AGAP1 in the PSD fractions (Yoshimura et al., 2004).

The localization of AGAP1 in axons and dendrites of cultured hippocampal neurons was determined by immunocytochemistry imaged by confocal fluorescence microscopy. We determined the upper and lower limits of protein colocalization by immunolabeling two proteins in the same compartment (secretogranin-3 and chromogranin-B), and two proteins in separate compartments (chromagranin-A and LAMP1), which yielded ~65% and ~7% colocalization, respectively. AGAP1 colocalized preferentially with two markers of dendritic compartment: MAP2 (52%) and the dendritic spine marker neurabin/spinophilin (35%), and slightly less with the postsynaptic marker PSD95 (17%; Figures 2B,C). In contrast, AGAP1 colocalized only slightly above nonspecific levels with the axonal marker Tau (17%) and the presynaptic markers SV2 (15%) and Syt1 (18%; Figures 2B,C). In summary, AGAP1 preferentially colocalizes with postsynaptic dendrites and dendritic spines.

**Figure 3:**
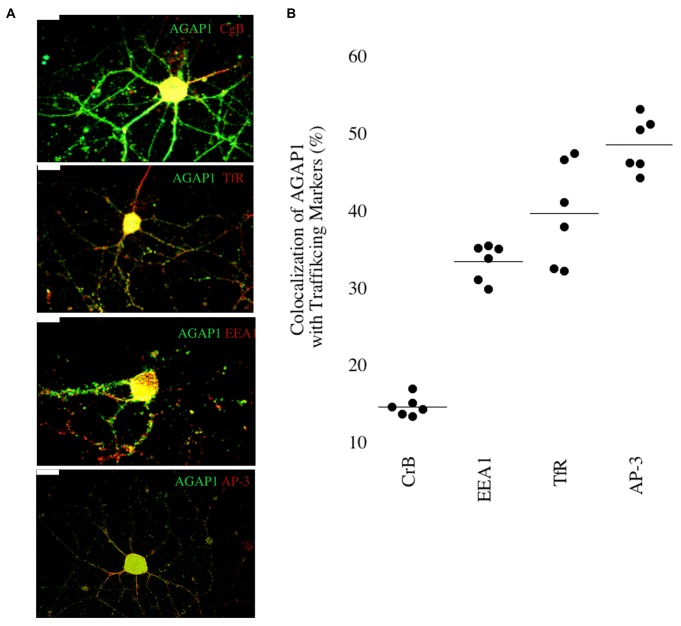
**Endogenous AGAP1 localizes to neuronal endosomal compartments. (A)** Immunocytochemical analysis of endogenous AGAP1 compared with vesicular compartment markers in 14 DIV dissociated rat E18 hippocampal neurons. White arrows indicate regions of colocalization. Green: AGAP1 and red: chromogranin B, adaptor protein 3 (AP-3), transferrin receptor, and early endosome antigen 1 (EEA-1) as indicated. Scale bar = 10 μm. **(B)** Quantification of colocalization within ROIs of endogenous AGAP1 with vesicular compartment markers. Background colocalization (~7%) is not subtracted. Data were quantified from three independent experiments (*n* = 10).

We then determined if AGAP1 localizes to endosomal compartments in dendrites in E18 hippocampal neurons. We compared AGAP1 localization with markers of vesicular compartments in dendrites (Figure 3). AGAP1 colocalized with transferrin receptors (40%), which label recycling endosomes, and with EEA1 (33%), which labels early endosomes (Figure 3; Parton et al., 1992; Prekeris et al., 1999; Park et al., 2004). AP-3 is a binding partner of AGAP1 that is expressed in both axons and dendrites, localizing to endosomes (Austin et al., 2002; Robinson, 2004; Salazar et al., 2004; Newell-Litwa et al., 2007). Indeed, AGAP1 colocalized strongly with AP-3 in hippocampal neurons (49%; Figure 3). Significantly lower colocalization was observed between AGAP1 and chromogranin B (15%), a marker of the regulated secretory pathway expressed in both axons and dendrites (Figure 3). Thus, consistent with data from non-neuronal cells, endogenous AGAP1 localizes predominantly to an AP-3 positive endosomal compartment in primary hippocampal neurons.

### Null Mutations of BLOC-1 Reduce The Expression Levels of AGAP1

AGAP1 associates with AP-3 and the BLOC-1 subunit dysbindin (Nie et al., 2003; Bendor et al., 2010). The BLOC-1 complex is an octameric complex containing dysbindin, pallidin, muted, cappucino, snapin, and BLOS1–3. In non-neuronal cells, BLOC-1 regulates trafficking from endosome lysosome-related organelles (Salazar et al., 2006, 2009; Li et al., 2007; Newell-Litwa et al., 2009; Ghiani and Dell’Angelica, 2011; Marks et al., 2013). In neuronal cells, BLOC-1 regulates vesicle trafficking from somatic endosomes to the synapse, as well as vesicle recycling at the synapse (Newell-Litwa et al., 2010; Larimore et al., 2011).

**Figure 4:**
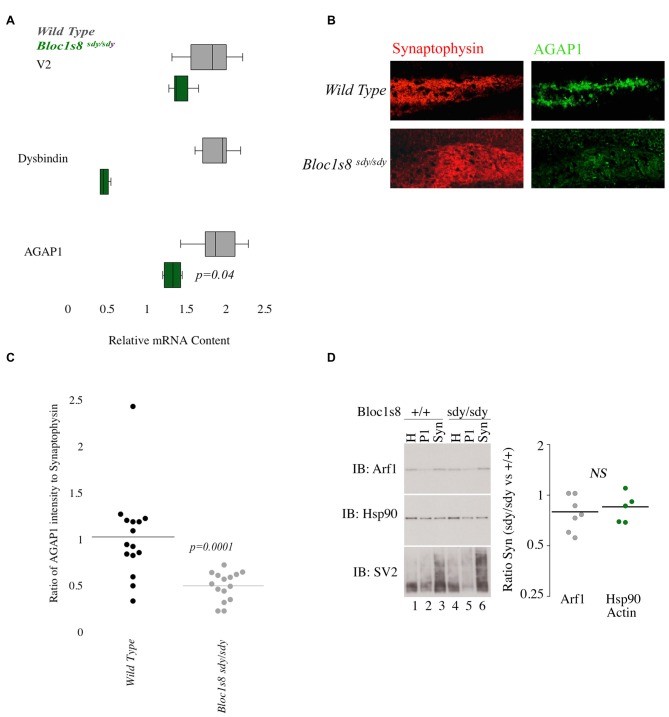
**AGAP1 is altered in the hippocampus of Biogenesis of Lysosome Related Organelles Complex-1 (BLOC-1) deficient mice. (A)** Relative mRNA levels in the hippocampus for wild type (WT; gray) and *Bloc1s8^sdy/sdy^* (green). Negative control Vamp2 (V2) and positive controls Dysbindin. No significant difference was measured for mRNA levels of V2. In *Bloc1s8^sdy/sdy^*, there was a significant decrease of dysbindin (*p* = 0.005). AGAP1 mRNA levels were significantly decreased in *Bloc1s8^sdy/sdy^* (*p* = 0.01). **(B)** Micrographs of the dentate gyrus at 20× for WT and *Bloc1s8^sdy/sdy^*. In red is synaptophysin and in green is AGAP1. **(C)** Quantification of the intensity levels of AGAP1 compared to synaptophysin in BLOC-1 deficient mice. AGAP1 intensity levels were significantly decreased in *Bloc1s8^sdy/sdy^* (*p* = 0.0001). **(D)** Arf1 levels in synaptosomes prepared in WT and *Bloc1s8^sdy/sdy^* mice. Hsp90 is a loading control, and SV2 is a synaptic vesicle marker to show enrichment of synaptosomes. No differences between WT and *Bloc1s8^sdy/sdy^* hippocampus for Arf1 levels as compared with loading controls actin (not shown) and Hsp90. *N* = 6 animals analyzed in three fractionations.

Since the AP-3 and BLOC-1 subunits *AP3B2* and *DTNBP1* are risk factors for ASD and SZ, respectively, we tested whether AGAP1 and BLOC-1 participate on a common signaling pathway by analyzing whether AGAP1 expression was sensitive to BLOC-1 deficiency. To this end, we first determined AGAP1 mRNA levels in the hippocampus of adult (P50) male *Bloc1s8^sdy/sdy^* (*sandy*) mice, which lack dysbindin, by qtPCR. Indeed, AGAP1 mRNA levels are significantly lower in the hippocampus of *Bloc1s8^sdy/sdy^* (*sandy*) mice than in that of age-matched WT mice (*p* = 0.04; Figure 4A). Next, we estimated AGAP1 protein levels by immunohistochemistry and confocal microscopy in the hippocampus of *Bloc1s8^sdy/sdy^* mice, as a ratio of synaptophysin levels, which are not altered in BLOC-1 mutants (Newell-Litwa et al., 2010; Larimore et al., 2011; Gokhale et al., 2012). Consistent with mRNA levels, AGAP1 fluorescence intensity was significantly lower in the dentate gyrus of *Bloc1s8^sdy/sdy^* mice (*p* = 0.0001; Figures 4B,C). Together, these data demonstrate that loss of dysbindin, a subunit of the SZ susceptibility factor BLOC-1, results in lower mRNA and protein levels of AGAP1.

Because AGAP1 is an Arf1 GAP, we wanted to determine the levels of Arf1 in the hippocampus of *Bloc1s8^sdy/sdy^* (*sandy*) mice. We prepared isolated synaptosomes from the hippocampus of WT and *Bloc1s8^sdy/sdy^* (*sandy*) mice. Synaptosomes were probed for Arf1, Hsp90 (control) and SV2 (synaptic vesicle protein 2) as a marker of synaptic vesicles. No significant difference was observed in the protein levels of Arf1 between WT and the *Bloc1s8^sdy/sdy^* (*sandy*) synaptosomes (Figure 4D).

### Null Mutations of Mecp2 Reduce mRNA but not Protein Levels of AGAP1

**Figure 5:**
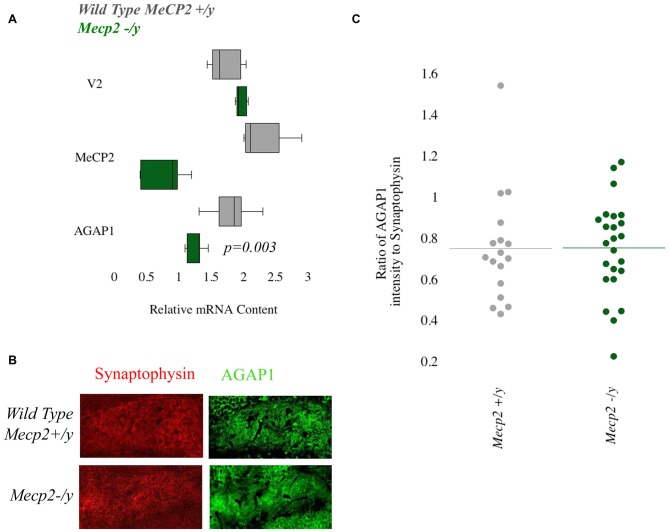
**Null mutations of Mecp2 reduce mRNA but not protein levels of AGAP1. (A)** Relative mRNA levels in the hippocampus for WT (gray) and *Mecp2^−/y^* (green). Negative control Vamp2 (V2) and positive controls Mecp2. No significant difference was measured for mRNA levels of V2. In *Mecp2^−/y^*, there was a significant decrease of Mecp2 (*p* = 0.005). AGAP1 mRNA levels were significantly decreased in *Mecp2^−/y^* (*p* = 0.003). **(B)** Micrographs of the dentate gyrus at 20× for WT and *Mecp2^−/y^*. In red is synaptophysin and in green is AGAP1. **(C)** Quantification of the intensity levels of AGAP1 compared to synaptophysin in *Mecp2^−/y^* mice. AGAP1 intensity levels were not significantly decreased in *Mecp2^−/y^* (*p* = 0.6).

We previously demonstrated that transcript and protein expression of the BLOC-1 complex subunit pallidin require *Mecp2* (Larimore et al., 2013). Mutations in *MECP2* result in Rett Syndrome, a neurodevelopmental disorder that is classified as an ASD. Based on this, we tested *Mecp2^+/y^* and *Mecp2^−/y^* to determine AGAP1 expression. First we determine AGAP1 mRNA levels in the hippocampus of adult (P50) mice. Results from qtPCR demonstrate reduced levels of AGAP1 mRNA in *Mecp2^−/y^* mice compared to controls (*p* = 0.003; Figure 5A). However, this reduction on AGAP1 mRNA levels did not translate into decreased AGAP1 protein expression as determined by immunohistochemistry and confocal microscopy in the hippocampus of *Mecp2^−/y^* mice. AGAP1 fluorescence intensity was unaltered in the dentate gyrus of *Mecp2^−/y^* mice (*p* = 0.6; Figures 5B,C). These data suggest that AGAP1 mRNA phenotypes are compensated at the protein expression level in *Mecp2^−/y^* mice. Our data argue that AGAP1 phenotypes are not a general feature downstream of genetic defects associated to neurodevelopmental disorders.

### AGAP1 Affects Transferrin Trafficking in Dendrites of Hippocampal Neurons

**Figure 6:**
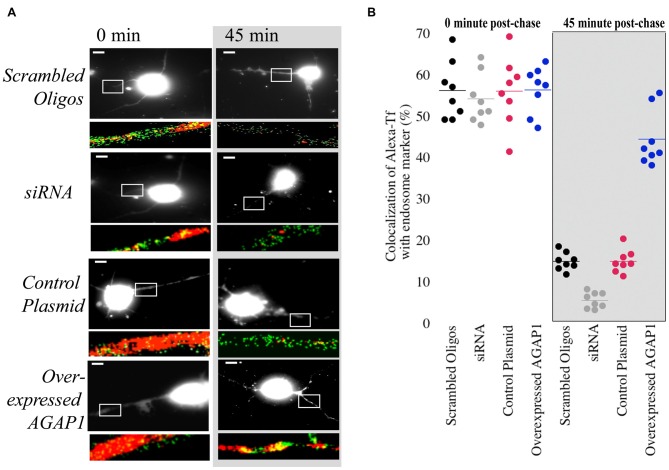
**Manipulation of AGAP1 in neuronal dendrites alters transferrin trafficking. (A)** Micrographs of 3 DIV E18 rat hippocampal neurons co-expressing eGFP and control plasmid, scrambled oligos, knock-down with siRNA, and overexpression of WT AGAP-1. Red: Alexa-transferrin (Alexa-Tf); Green: Rab11. White box outlines a ROI enlarged beneath the micrograph. Right panels: transferrin uptake was determined after 30 min (0 min). Left panels: recycling was assessed after 45 min following incubation with unlabeled transferrin (45 min). Scale bar = 10 μm. **(B)** Quantification of percent colocalization within ROIs of Rab11 puncta with Alexa-Tf. Data were quantified from three independent experiments (*n* = 10). At the 0 min time point no significant difference was observed between the colocalization of Rab11 and Alexa-Tf in scrambled vs. siRNA for AGAP1 (*p* = 0.6) and control plasmid vs. overexpressed AGAP1 (*p* = 0.9). At the 45 min time point, colocalization of Rab11 and Alexa-Tf was significantly different in scrambled vs. siRNA for AGAP1 (*p* = 0.0002) and for control plasmid vs. overexpressed AGAP1 (*p* = 0.0002).

Overexpression of AGAP1 in non-neuronal cells results in the accumulation of transferrin in large punctate structures (Nie et al., 2002, 2005). In hippocampal neurons, transferrin is endocytosed into dendrites via transferrin receptors, and traffics through the recycling endosomal compartment that contains Rab11 (Parton et al., 1992; Prekeris et al., 1999; Park et al., 2004, 2006). Based on the significant colocalization of AGAP1 with transferrin receptors, we tested whether up or down regulation of AGAP1 affects transferrin trafficking in dendrites of hippocampal neurons by imaging the trafficking of Alexa-transferrin (Alexa-Tf). After 30 min of bath application of Alexa-Tf, we chased cells with unlabeled transferrin to determine if Alexa-Tf exits recycling endosomes properly. After 30 min at 37°C, Alexa-Tf showed a punctate pattern colocalizing extensively with the recycling endosome marker Rab11 (Figure 6). Immediately after the chase with unlabeled transferrin, 57% of Rab11 positive endosome puncta were also positive for Alexa-Tf (Figure 5), demonstrating Alexa-Tf persistence in the recycling endosome. After 45 min of chase with unlabeled transferrin, there was a significant reduction in the intensity of Alexa-Tf, suggesting that transferrin had trafficked out of recycling endosomes to either the plasma membrane or cell body (Figure 6).

In control neurons and neurons overexpressing AGAP1, there were no significant differences in the colocalization of Alexa-Tf with Rab11-labeled recycling endosomes immediately after the chase with unlabeled transferrin (Figure 6). In contrast, overexpressing AGAP1 increased the levels of Alexa-Tf that remained in the Rab11-positive compartment after 45 min of chase (Figure 6), suggesting a slower rate of Alexa-Tf trafficking out of recycling endosomes. When AGAP1 was knockdown with siRNA, the uptake of Alexa-Tf was unaffected immediately after the chase (Figure 6). However, the levels Alexa-Tf that remained in the Rab11-positive compartment after 45 min of chase were lower in AGAP1 siRNA-expressing neurons compared to control cells (Figure 6), suggesting that down regulation of AGAP1 increases the rate of transferrin recycling in dendrites. These gain and loss-of-function data support a role for AGAP1 in endosomal trafficking in dendrites of hippocampal neurons.

### AGAP1 Modulates the Morphology of Dendritic Spines

Disruption of endosomes or altering dysbindin levels affects the morphogenesis of dendritic spines (Park et al., 2006; Jia et al., 2014). Therefore, we tested whether AGAP1 overexpression or siRNA-mediated knockdown affected dendritic spines of CA1 and CA3 pyramidal neurons in organotypic cultures of hippocampal slices, which preserve neuronal integrity and hippocampal circuitry, developing *in vitro* similarly as *in vivo* (Lo et al., 1994; Gähwiler et al., 1997; Alonso et al., 2004). Neurons in slice cultures were cotransfected with eYFP and either WT AGAP1 or siRNA for AGAP1. CA1 and CA3 pyramidal cells were identified by their characteristic morphology and anatomical location within an hippocampal slice. Previous studies have shown that over 90% of neurons in slice cultures transfected with two cDNA plasmids by biolistic gene transfer express both proteins (Moore et al., 2007; Chapleau et al., 2009).

**Figure 7:**
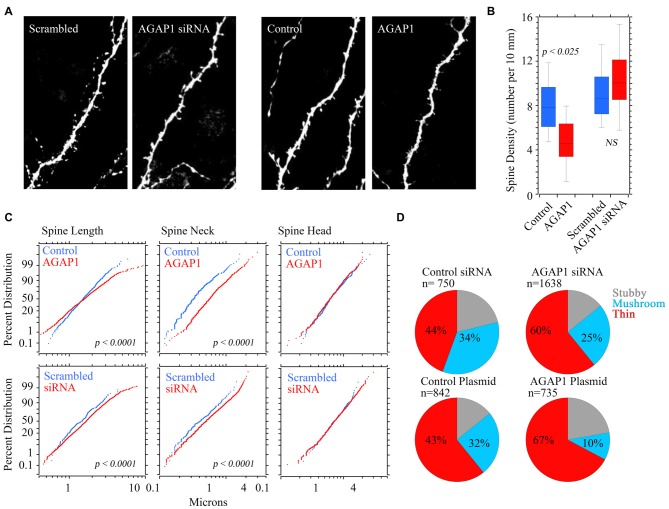
**Manipulation of AGAP1 levels alters spine density and morphology in CA1/CA3 neurons in organotypic hippocampal slice cultures. (A)** Representative dendritic segments of CA1/CA3 pyramidal neurons from organotypic slice cultures transfected with yellow fluorescent protein (YFP) and control plasmid, scrambled oligos, siRNA or overexpression of WT AGAP1. Scale bar = 2 μm. **(B)** Quantification of spine density in each condition. Data were quantified from three independent experiments (*n* = 8–12 dendrites). Significant decrease in spine density was observed in AGAP1 overexpressing slice cultures (*p* = 0.02). No significant change was observed in spine density in siRNA treated slice cultures (*p* = 0.5). **(C)** Quantification of spine length, neck size, and spine head diameter in each condition. Data were quantified from three independent experiments (*n* = 8–12 dendrites). There was significant increase in spine length (*p* < 0.0001) by either up or down regulation of AGAP1. There was significant increase in the diameter of spine neck (*p* < 0.0001) by either up or down regulation of AGAP1. No significant change in spine head diameter was observed. **(D)** Quantification of spine morphology in each condition. Data were quantified from three independent experiments, three separate cells per condition, 8–12 pieces of dendrite per cell. *N* = the number of spines counted. Thin spines increased to ~60% after siRNA AGAP down-regulation (*p* < 10^−12^ Fisher’s exact test) or to ~67% after overexpression of AGAP1 (*p* < 10^−29^ Fisher’s exact test).

Pyramidal neurons that overexpressed WT AGAP1 had significantly lower dendritic spine density, with an average 40% reduction (4.7 spines/10 μm in AGAP1 vs. 8.0 spines/10 μm in control; Figure 7, *p* = 0.02). On the other hand, siRNA-mediated AGAP1 knockdown caused a change in spine density that did not reach statistical significance (10.6 spines/10 μm in siRNA vs. 8.0 spines/10 μm in control; Figure 7, *p* = 0.08).

We blindly quantified spine morphological changes after genetic manipulation of AGAP1 activity. We either expressed data as absolute values in spine length; neck, and head size (Figure 7C) or we categorize spines as spiny, stubby or mushroom shaped spines according to Boda and Pozzo-Miller (Tyler and Pozzo-Miller, 2003; Boda et al., 2004; Figure 7D). Spine length and neck size were significantly increased by either up or down regulation of AGAP1 (Figure 7C). Next, we compared the proportions of spine classes according to a classification scheme of three morphological types: stubby, mushroom and thin (Tyler and Pozzo-Miller, 2003; Boda et al., 2004). Both siRNA-mediated AGAP1 knockdown and AGAP1 overexpression altered the proportions of spine types from predominately mushroom spines to primarily immature spines (Figure 7D). The proportion of thin spines increased to ~60% after AGAP knockdown (*p* = 5.55 × 10^−13^, Fisher’s exact test), and to ~67% after AGAP1 overexpression (*p* = 5.30 × 10^−13^; Figure 7). These data demonstrate that AGAP1 levels modulate both dendritic spine density and the proportion of morphological spine types in hippocampal pyramidal neurons.

## Discussion

Genes encoding dysbindin and AGAP1 have been implicated as risk factors for SZ and ASD. Moreover, here we demonstrate that deficiencies in AGAP1 generate dendritic spine deficits characteristic of developmental disorders associated with intellectual disabilities (Penzes et al., 2011, 2013; Seshadri et al., 2013). We describe the subcellular localization and consequences on neuronal morphology of altering the levels of AGAP1, the first Arf1-GAP to be characterized in developing neurons. AGAP1 is expressed in the cortex, thalamus, hippocampus, and brainstem. The results presented in this study indicate that AGAP1 is a regulator of endosomal trafficking in neurons, and a negative regulator of dendritic spine formation and maturation. We also demonstrate that loss of the BLOC-1 subunit dysbindin alters AGAP1 mRNA levels and immunoreactivity in the dentate gyrus. We postulate that BLOC-1 resides upstream of AGAP-1 to regulate endocytic recycling in dendritic spine endosomes. This BLOC-1-AGAP1 pathway is insensitive to genetic defects in Mecp2, indicating that neurodevelopmental disorder genes such as Mecp2 and DTNBP1 use different mechanisms to control spine morphology.

AGAP1 localizes to axons, dendrites, dendritic spines and synapses in developing hippocampal neurons, and during prenatal and early postnatal brain development. This period in development corresponds to a phase of differentiation of axons and dendrites, especially their growth and arborization, formation of dendritic spines and synapses in the cortex and hippocampus (Gaarskjaer, 1981; Melloni and De Gennaor, 1994), suggesting that AGAP1 participates in these developmental events. In agreement with this model, siRNA-mediated AGAP1 knockdown resulted in higher dendritic spine density, and a higher proportion of immature thin dendritic spines (Type-3), while AGAP1 overexpression caused exactly the opposite results (lower density of spines, more thin spines). Previous studies reported that loss-of-function of dysbindin decreased spine density, a phenotype reminiscent of the AGAP1 gain-of-function phenotype (Jia et al., 2014); this result was unexpected because dysbindin loss leads to lower levels of AGAP1. However, there are some differences in experimental preparations: Jia et al. (2014) utilized hippocampal neurons isolated from E18 to E19 *Bloc1s8^sdy/sdy^* (*sandy*) mice, which lacked dysbindin throughout their development. Here, we acutely downregulated AGAP1 levels in organotypic cultures of hippocampal slice from P7 to 10 rats, where neurons had developed normally until the time of AGAP1 siRNA expression. These differences in the developmental time of AGAP1 deletion may account for the apparent discrepancy in the numerical density of dendritic spines. Regardless, both AGAP1 and BLOC-1 regulate an endosomal pathway that participates in the formation and morphological maturation of dendritic spines during brain development.

Endogenous AGAP1 colocalizes extensively with markers of early and recycling endosomes in dendrites (Nie et al., 2002, 2005). Here, we studied how AGAP1 expression levels affect endosomal trafficking in dendrites. Down regulation of AGAP1 enhanced the rate of transferrin trafficking in dendrites, while AGAP1 overexpression caused the exact opposite result. Endosomal trafficking in dendrites is necessary for several functions, including activity-dependent synaptic plasticity (e.g., long-term potentiation, LTP) and regulation of dendritic spine morphology. Previous studies demonstrated that a collapse of recycling endosomes reduces spine density in an activity-dependent manner (Park et al., 2006). In addition, recycling endosomes located at the base of dendritic spines regulate spine growth during LTP (Blanpied and Ehlers, 2004; Park et al., 2004, 2006; Blanpied et al., 2008). Based on these combined observations, the possibility that AGAP1 participates in neuronal differentiation by regulating endosomal trafficking is intriguing. Here, we propose a mechanism whereby dysbindin regulates AGAP1 expression to modulate spine density and morphology. Collectively, these data indicate that fine-tuned expression of AGAP1 is necessary the formation and maturation of dendritic spines during brain development, suggesting that AGAP-dependent mechanisms play a role in the pathogenesis of neurodevelopmental disorders.

## Author Contributions

MA, RC, and KSS contributed equally to the manuscript and are co-first authors. All authors contributed to the manuscript.

## Conflict of Interest Statement

The authors declare that the research was conducted in the absence of any commercial or financial relationships that could be construed as a potential conflict of interest.

## Footnotes

^1^https://www.cog-genomics.org/software/stats

^2^http://www.physics.csbsju.edu/stats/KS-test.n.plot_form.html

^3^http://mouse.brain-map.org/gene/show/110324

^4^http://www.ncbi.nlm.nih.gov/pubmed/23193282
